# Enhanced Seamless Video Fusion: A Convolutional Pyramid-Based 3D Integration Algorithm

**DOI:** 10.3390/s24061852

**Published:** 2024-03-14

**Authors:** Yueheng Zhang, Jing Yuan, Changxiang Yan

**Affiliations:** 1 Changchun Institute of Optics, Fine Mechanics and Physics, Chinese Academy of Sciences, Beijing 100045, China; zhangyueheng17@mails.ucas.ac.cn (Y.Z.); 15543665143@163.com (J.Y.); 2 University of Chinese Academy of Sciences, Beijing 100049, China; 3 Center of Materials Science and Optoelectrics Engineering, University of Chinese Academy of Sciences, Beijing 100049, China

**Keywords:** seamless editing, convolutional pyramid, three-dimensional Poisson equations

## Abstract

Video fusion aims to synthesize video footage from different sources into a unified, coherent output. It plays a key role in areas such as video editing and special effects production. The challenge is to ensure the quality and naturalness of synthetic video, especially when dealing with footage of different sources and qualities. Researchers continue to strive to optimize algorithms to adapt to a variety of complex application scenarios and improve the effectiveness and applicability of video fusion. We introduce an algorithm based on a convolution pyramid and propose a 3D video fusion algorithm that looks for the potential function closest to the gradient field in the least square sense. The 3D Poisson equation is solved to realize seamless video editing. This algorithm uses a multi-scale method and wavelet transform to approximate linear time. Through numerical optimization, a small core is designed to deal with large target filters, and multi-scale transformation analysis and synthesis are realized. In terms of seamless video fusion, it shows better performance than existing algorithms. Compared with editing multiple 2D images into video after Poisson fusion, the video quality produced by this method is very close, and the computing speed of the video fusion is improved to a certain extent.

## 1. Introduction

In recent years, as short videos have become more and more popular in people’s lives, people have started to be interested in shooting and editing their own short videos. In order to form short videos with their own style, people need to use various editing tools, and they need to generate videos that are as natural and realistic as possible.

Common video editing tools can only perform simple and common editing tasks, such as cutting and pasting video clips, resizing, colour correction, making simple transitions, and generating titles. These are far from what users need. Assuming that users need to split out certain features from one video sequence and paste them into another, the challenge of such complex video editing tasks is associated with two limitations:

(1) Spatial consistency: the imported objects should blend seamlessly with the background. As a result, pixel substitution creates visible seams, which is problematic.
(2) Temporal coherence: consecutive frames should show smooth transitions. Therefore, frame-by-frame editing that results in visual flickering is inappropriate.

If either of these two aspects is violated, certain artefacts (e.g., flickering) may cause distortion in the generated video. The goal of this paper is to propose a new algorithm, which can realize seamless video fusion, make the video smooth in the time domain, and help to avoid artifacts, flickers, and other defects.

Poisson image editing, as an algorithm for reconstruction based on a gradient field, is a well-known image fusion algorithm in the field of image processing with natural boundary transition. This algorithm can achieve seamless editing of images, so we use the Poisson image editing algorithm as the basis of our video editing algorithm. Since Pérez proposed Poisson image editing [1], many improvements and applications have been introduced with respect to this algorithm.

Jia et al. [2] presented a user-friendly system called “drag-and-drop pasting” for seamless image composition. It addresses challenges in Poisson image editing, where precise boundary delineation is crucial. To simplify this process, the authors proposed a new objective function and a shortest closed-path algorithm to optimize the boundary conditions automatically. Georgiev [3] described a new theoretical framework for image processing and vision, where images are viewed as graphs on a fiber bundle. This approach accommodates human visual perception, handles illumination changes seamlessly, and explains visual illusions like simultaneous contrast. It facilitates various image processing tasks, such as Poisson image editing, inpainting, and HDR compression. Sadeghi et al. [4] studied Poisson local color correction for image stitching. A new method for seamless image stitching was presented. To evaluate and compare the proposed method with competing ones, a large image database consisting of more than two hundred image pairs was created.

Jeschke et al. [5] presented a new Laplacian solver for minimal surfaces—surfaces having a mean curvature of zero everywhere except at some fixed (Dirichlet) boundary condition. Two main contributions were made: First, a robust rasterization technique was provided to transform continuous boundary values (diffusion curves) to a discrete domain. Second, a variable stencil size diffusion solver was defined that could solve the minimal surface problem. Tanaka et al. [6] introduced a modified Poisson problem algorithm for better color preservation in seamless image cloning. The proposed algorithm optimizes the entire image region, introducing a color-preserving parameter for precise control over color adaptation levels. The improved color preservation offers a more efficient and effective solution for image cloning compared to traditional methods.

Shibata et al. [7] presented a novel image fusion algorithm for a visible image and a near-infrared (NIR) image. The gradient information is fused and the output image is constructed by Poisson image editing, which preserves the gradient information of both images. They presented a novel unified image fusion framework based on an application-adaptive importance measure; however, it suffers from two main problems: color bleeding and bleeding artifacts. Afifi et al. [8] presented a modified Poisson blending technique (MPB) for image cloning. The technique addresses the problems of color bleeding and bleeding artifacts that occur in traditional Poisson image editing. They also provided background information concerning gradient domain processing and discussed related work in image blending techniques. Martino et al. [9] reviewed the contributions that have been made since Poisson image editing was proposed in 2003. They explore the theory of Poisson image editing and its evolution since its introduction in 2003. The integration problem of processing and integrating a given vector field is addressed theoretically and numerically. Two numerical implementations are provided: one using discrete differential operators and the other based on Fourier transform properties. The paper also examines related work and applications.

Poisson image editing is the process of creating a new image *f* seamlessly. Some gradient domain methods obtain the optimal pixel value by solving the Poisson equation [10], and fuse the source image and the target image while retaining the gradient information of the source image. The method solves a Poisson equation according to the boundary conditions specified by the user and realizes continuity on the gradient domain so as to achieve seamless fusion at the boundary. The Poisson equation is solved by transforming it into a large sparse linear system:

(1) Ax=b

Assume that Ω is the selected region on the source image with the boundary ∂Ω. Pérez et al. set a new intensity value *f* for all the pixels in the region Ω that minimize the difference between the gradient of f(∇f) and the vector field ν. This method assigns the intensity values of *f* at the boundary of the region ∂Ω to the values at the boundary of the destination image $f^{*}$ ($f=f^{*}$ over ∂Ω). This process can be expressed by Equation (2):

(2) $$min_{f}{\;\int \int \;}_{\Omega }{\left|\nabla f-\nu \right|}^{2}\;\mathrm{with}\;f{|}_{\partial \Omega }=f^{*}{|}_{\partial \Omega }$$

The solution is the only solution of Laplace’s equation [11,12,13] with Dirichlet boundary conditions,

(3) $$\Delta f=\mathrm{div}\;\nu \;\mathrm{over}\;\;\Omega \;\;\mathrm{with}\;f{|}_{\partial \Omega }=f^{*}{|}_{\partial \Omega }$$

**Figure 1:**
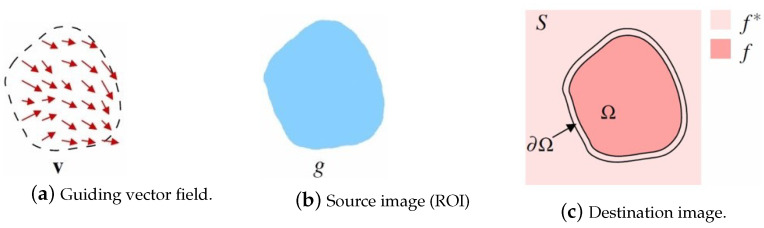
Guided interpolation notations. The process of Poisson image editing can be described as an unknown function f which interpolates in domain Ω the destination function $f^{*}$, under the guidance of the vector field **v**.

Set the guidance field to be the gradient of the source image: v=∇g. Thus, Equation (3) becomes

(4) $${\Delta f=\Delta g\;\mathrm{over}\;\Omega \;\;\mathrm{with}\;f|}_{\partial \Omega }=f^{*}{|}_{\partial \Omega }$$

where Δ is the Laplacian operator. The discrete solution to the previous Equation (4) is equal to the solution of the linear system of Equation (2), where *A* is an (n×n) matrix and *n* is the number of the unknown pixels in the selected region. The solving process is shown in Figure 1.

Because the size of matrix *A*, often related to the size of the region of interest(ROI), could be of the order of thousands to millions as well as the unknowns, some iterative methods have been proposed to solve it. These include the Jacobi iteration method, the Gauss–Seidel method, the successive over-relaxation (SOR) method, and other classical methods. Many people have sought to improve these classical methods to reduce the number of iterations and improve the efficiency of the operation.

In 2012, Akhir, M Kamalrulzaman et al. [14] combined the four-point explicit decoupling group (EDG) iteration method with the Gauss–Seidel (GS) iteration method to solve linear systems discretely generated by a finite difference scheme using the second-order central difference, which can also be used to solve the Poisson equation. In 2017, Eng Jeng Hong et al. [15] focused on solution of the Poisson equation in image mixing, and evaluated and compared the Jacobi, Gauss–Seidel, and SOR iterative methods. Through evaluation of the number of iterations and the calculation time, the results showed that the SOR iterative method was more effective and efficient in solving the Poisson image mixing problem. In 2017, these authors proposed an improved MSOR iterative method for the Poisson image mixing problem, and through comparison experiments with other iterative methods proved that the MSOR method produces better solution results in the field of image editing, showing higher iteration efficiency with a shorter computation time [16]. In 2018, they further developed the SOR algorithm and proposed the 4-EDGSOR iterative method to solve the Poisson image mixing problem, and demonstrated the advantages of this method compared with other methods, in terms of time, number of iterations, and image quality, through numerical experiments [17].

In contrast to improving the efficiency of the algorithm to solve the equation, some researchers have also introduced other algorithms to transform the form of the Poisson equation to improve the efficiency of image fusion. In 2009, Farbman et al. [18] introduced an approach based on mean-value coordinates (MVC) for seamless cloning operations, which has advantages in terms of speed, ease of implementation, small memory footprint, and parallelism. Compared to the traditional Poisson equation solving method, it has advantages in dealing with large-area real-time cloning and interactive cloning of video streams, and shows feasibility and flexibility in application programs. In 2010, Xie et al. [19] proposed an optimized mean cloning technique and combined it with a new seamless video synthesis framework to solve the problems of smears and discoloration in traditional methods. Through the introduction of a contour flow propagation model and interactive tool, the challenge of low efficiency of a trimap propagation model was solved successfully. In 2011, Farbman et al. [20] proposed a fast convolutional numerical approximation method based on large support filters, which realizes multi-scale transformation through numerical optimization of small kernel design, providing an efficient solution for signal processing. This method shows superiority in multiple application scenarios and performs better than existing methods in tasks such as gradient field integration, image processing, and data interpolation.

In view of the lack of simple real-time studies on seamless video fusion algorithms, this paper proposes a seamless video synthesis technique for gradient domain 3D integration. This is a natural extension of the spatio-temporal space based on the idea of Poisson image editing proposed by Pérez et al. [1]. Wang et al. [21] proposed a 3D video integration algorithm, which found a potential function whose gradient field was closest to the resulting gradient field in the sense of least squares. The video was reconstructed by solving a 3D Poisson equation.

Instead of processing video clips frame-by-frame, this approach treats the entire video as a 3D cube in space-time space. In order to improve the processing speed, we propose a new fast convolution numerical approximation method, which adopts the diagonal multigrid proposed by Farbman [20], including a multi-scale scheme, which is transformed by wavelet to calculate the linear time approximation. To achieve an approximation for a particular large target filter, we first use numerical optimization to design a set of small cores, which are then used to perform the analysis and fusion steps of our multi-scale transformations.

The analysis results for fusion video quality and fusion speed show that the proposed video fusion method is effective, ensuring the quality of the fusion video, greatly reducing the computational complexity, and avoiding the requirement for more complex processing steps. In addition, multi-dimensional processing technology is combined to achieve acceleration so as to achieve rapid fusion of a 1 million pixel video area on the current mainstream GPU.

## 2. Methods

The current gradient field method [1,22,23,24,25] can be viewed as a modification of two-dimensional integrals for color allocation. The luminance scale, moving blur, and the image dependence index are considered in the integration process. However, applying this method directly to video processing frame-by-frame results in flickering brightness and color and a lack of time consistency.

To solve this problem, we propose a new method, which is to convert the video into a three-dimensional cube and process it by modifying the three-dimensional integral of the three-dimensional gradient field. Compared with the convolution approximation of the free space Green’s function [26], our method introduced in Section 2.2 is more accurate and is easy to implement. Although we have demonstrated the effectiveness of this approach in many applications, the theoretical analysis of these questions remains incomplete, providing interesting directions for future work to explore.

### 2.1. Image Pyramid

An “image pyramid” is a multi-scale image representation in which the bottom layer of the image structure is the original image and each layer up is obtained by performing a series of downsampling and filtering operations on the next layer. Each layer of the pyramid is obtained by blurring and downsampling the previous layer, thus preserving the features of the image at different scales.

Image pyramids are widely used in the field of computer vision and image processing for feature extraction, target detection, image fusion, image compression, image pyramid matching, and other tasks. They have an important role in many image processing and computer vision applications due to their ability to preserve the features of an image at different scales and to handle scale variations efficiently.

There are two common types of image pyramids which are described below.

#### 2.1.1. Gaussian Pyramid

This is a series of images obtained by successive downsampling of the maximum resolution image at the bottom. The image at the bottom has the highest resolution, and the image resolution decreases as you go up.

The downsampling process of the Gaussian pyramid [27,28] is as follows:

(1) For a given image, a Gaussian smoothing process is performed first; that is, a convolution check image with a size of 5×5.
(2) Then, the image is sampled again, the even rows and even columns in the image are removed, and a picture is then obtained.
(3) Loop (1) and (2) operations on this picture to obtain the Gaussian pyramid.

**Figure 2:**
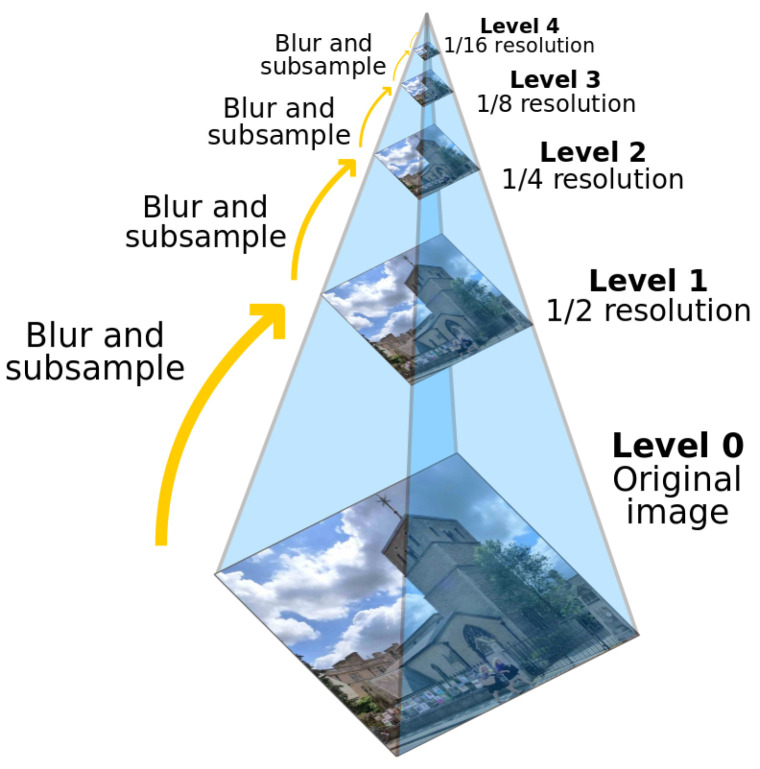
Schematic representation of the model of the Gaussian pyramid.

As can be seen from Figure 2, the image obtained by a cycle is the image of $G_{\left(i+1\right)}$. The entire pyramid is calculated by iterating through the input image $G_{i}$ (the original image). At the same time, it is also clear that downsampling will gradually discard the information of the image. This is the downsampling operation of the image; that is, the image is reduced. The downsampling process causes spectrum compression and may result in aliasing.

The upsampling process of the Gaussian pyramid is as follows:

(1) The size of the image is doubled in each direction and the new rows and columns are filled with zeros.
(2) The same kernel (multiplied by 4) is convolved with the enlarged image to obtain an approximation of the “added pixels”

The resulting image is an enlarged image, but it is somewhat blurred compared to the original image because some information has been lost in the process of zooming, and these data form the Laplacian pyramid [27,29].

#### 2.1.2. Laplacian Pyramid

The Laplacian pyramid is used to reconstruct the upper unsampled image from the lower image of the pyramid in digital image processing, i.e., prediction of residuals, to maximize the recovery of the image for use with the Gaussian pyramid.

The simple difference between the two is that the Gaussian pyramid is used to downsample the image. Note that downsampling is actually sampling from the bottom of the pyramid up with reduced resolution, which is contrary to the concept of pyramids as we understand it. The Laplacian pyramid, on the other hand, reconstructs the image from the base of the pyramid by upsampling the image.

**Figure 3:**
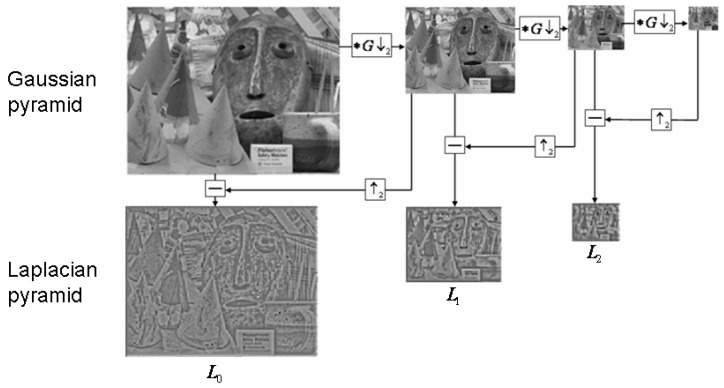
Schematic representation of the model of the Laplacian pyramid.

Gaussian pyramid downsampling is first performed with Gaussian blur and then downsampling. The image only contains low-frequency information except for the resolution reduction. The Laplacian pyramid firstly subtracts the source image (the Gaussian pyramid downsamples the low-frequency image), then enlarges the image to extract the high-frequency residual, samples the source image by Gaussian pyramid downsampling, and then reconstructs (restores) the image by upsampling. The process is shown in Figure 3.

The Gaussian pyramid subsampling with Gaussian blur before subsampling only contains low-frequency information except for the resolution reduction. The Laplacian pyramid extracts the high-frequency residual from the source image by first subtracting the image after downsampling (low-frequency Gaussian pyramid downsampling) and then enlarging the image, which helps to reconstruct the image after Gaussian pyramid downsampling and then upsampling (recovery).

**Figure 4:**
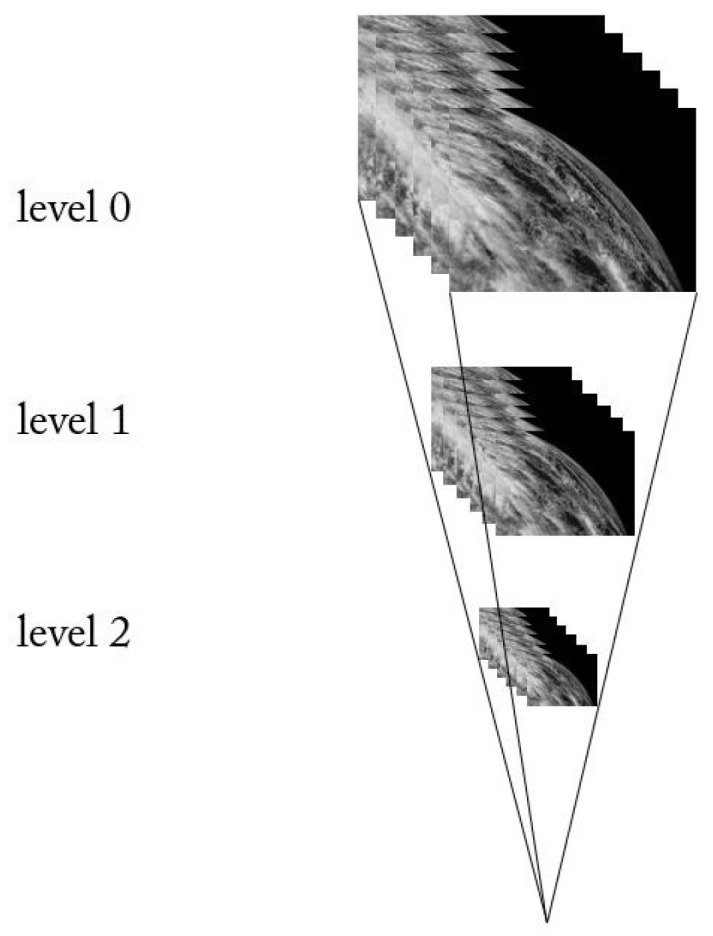
Schematic representation of the model of an image cube pyramid.

In this experiment, multiple frames of images are assembled into image cubes. The image cubes corresponding to two videos need to be downsampled and upsampled during the video fusion process, as Figure 4 shows. It is notable that our synthesis is not intended to reverse the analysis and reproduce the input signal, unlike most sub-band architectures. Instead, the combined operations of the up- and downsampling transformations are used to approximate the results of some particular linear translation-invariant filtering operations applied to the input. The result of this operation is the same as that applied to the input.

### 2.2. 3D Video Integration

Our task is to generate a new video *I* by fusing two videos into one, whose gradient field is closest to the modified gradient, *G*. One natural way to achieve this is to solve the equation:

(5) ∇I=G

Since we only consider the gradient field, we use a formula similar to Fattal et al. [22] and extend it to three dimensions by considering both the spatial and temporal gradients. Our task is then to find a potential function *I*, whose gradient is closest to *G* in the least square sense by searching the space of all 3D potential functions, i.e., minimizing the following integral in 3D space (hence, the reference to the 3D video integral in a follow-up):

(6) f=min∫∫∫F(∇I,G)dxdydt,

where,

(7) $$\begin{array}{cc} F\left(\nabla I,G\right) & ={∥\nabla I-G∥}^{2}\\ & ={\left(\frac{\partial I}{\partial x}-G_{x}\right)}^{2}+{\left(\frac{\partial I}{\partial y}-G_{y}\right)}^{2}+{\left(\frac{\partial I}{\partial t}-G_{t}\right)}^{2} \end{array}$$

According to the variational principle, a function *F* that minimizes the integral must satisfy the Euler–Lagrange equation [30]:

(8) $$\frac{\partial F}{\partial I}-\frac{d}{dx}\frac{\partial F}{\partial I_{x}}-\frac{d}{dy}\frac{\partial F}{\partial I_{y}}-\frac{d}{dt}\frac{\partial F}{\partial I_{t}}=0$$

We can then derive a 3D Poisson Equation:

(9) $$\nabla^{2}I=\frac{\partial^{2}}{\partial x^{2}}+\frac{\partial^{2}}{\partial y^{2}}+\frac{\partial^{2}}{\partial t^{2}}$$

and ∇·G is the divergence of the vector field *G*, defined as:

(10) $$\nabla \cdot G=\frac{\partial G_{x}}{\partial x}+\frac{\partial G_{y}}{\partial y}+\frac{\partial G_{t}}{\partial t}$$

**Figure 5:**
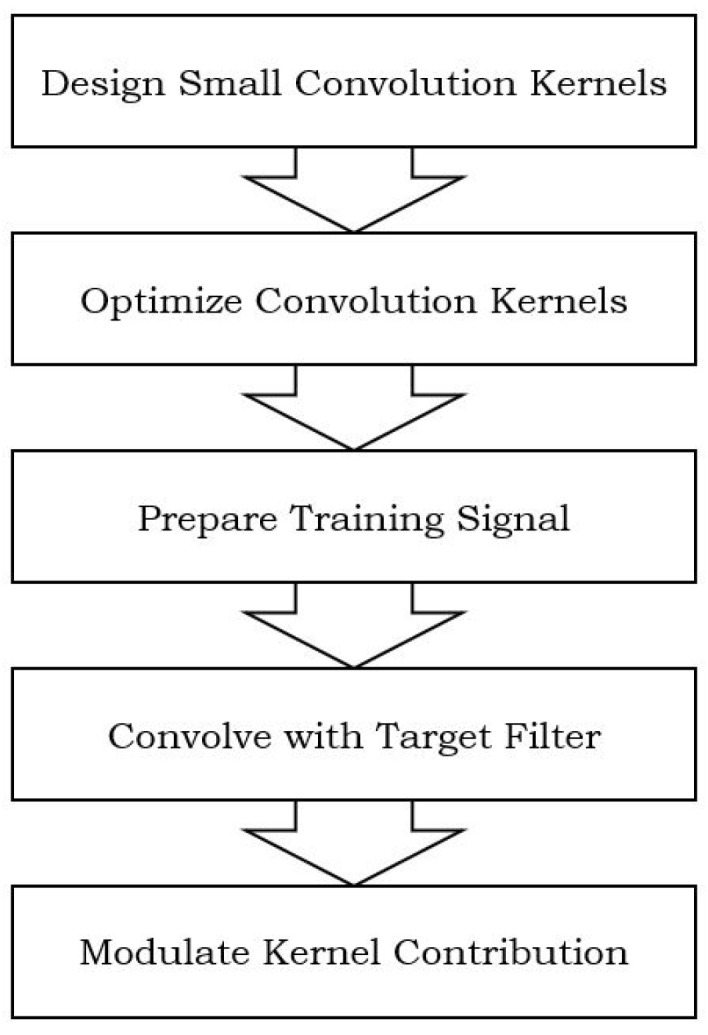
Flowchart describing the workflow of the seamless video cloning procedure.

The flowchart describing the workflow of the seamless video cloning procedure based on convolution pyramids is shown in Figure 5:

(1) Designing Kernels: The process starts with designing a set of small kernels through numerical optimization to approximate convolutions with large support filters efficiently.
(2) Optimizing the Kernels: The designed kernels are optimized to achieve an adequate approximation across the entire domain, ensuring translation invariance in the resulting transform.
(3) Training Signal Preparation: The training signal is set to a sum of randomly translated and scaled delta functions to prepare for the optimization process.
(4) Convolution with Target Filter: The training signal is convolved with the target Gaussian filter to obtain the desired convolution result.
(5) Modulating Kernel Contribution: To enhance the approximation quality, the contribution of certain kernels at each level is modulated by scalar weights to improve the overall performance.

By following this workflow, the seamless image cloning procedure based on convolution pyramids leverages the efficient approximation capabilities of the multi-scale scheme to achieve superior results in image cloning tasks.

In Algorithm 1, the workflow is explained more intuitively:
**Algorithm 1** Convolution Pyramid Algorithm**Input:** Trained filter kernel *w*, the target and source videos;
**Output:** Result video
  1:Building filter kernels: $H=[w_{0},w_{1},w_{2},w_{1},w_{0}]$, $G=[w_{2},w_{3},w_{4}]$$h_{1}=H^{T}H$, $h_{2}=w_{3}\cdot h_{1}$, $g=G^{T}G$  2:**for** each pixel *j* in the source and target image cubes **do**  3:    Set the non-boundary regions of the error image a(i,j) to zero;  4:    Apply the characteristic function chi to a(i,j), setting the values in non-boundary regions to zero;  5:    Filter a(i,j,k) and chi using the convolution pyramid filters, resulting in Ierf(i,j,k) and Ichi(i,j,k);  6:    Compute the corrected value $temp\left(i,j,k\right)=\frac{Ierf(i,j,k)}{Ichi(i,j,k)}+src\left(i,j,k\right)$, where src(i,j,k) is the value of the current pixel in the source image;  7:    Assign the corrected value to the corresponding position in the target image tr(i,j,k);  8:**end for**  9:Generate the result:10:Merge the corrected channel results to form the final result res;11:Overlay the result onto the corresponding positions in the target image;12:The resulting video is obtained from the pyramid reconstruction.


#### 2.2.1. 3D Discrete Poisson Solver

In image processing and computer graphics, large linear translation-invariant (LTI) filters play a key role. These filters are widely used in image processing, covering operations such as low-pass and high-pass filtering, and measuring the filter response. In addition to common tasks, some less obvious tasks can also be performed by large LTI filters, such as image reconstruction through integral gradient fields, or using the photosynovial membrane to fit the boundary value set [1,31], and interpolation dispersion data [32].

Although convolution is a direct means of applying LTI filters, it is computationally expensive, requiring $O(n^{2})$ operations to convolve an n-pixel image with a corresponding size kernel. To solve this challenge, the fast Fourier transform becomes a more efficient option, which can perform O(nlogn) operations in the periodic domain. In addition, for special cases, there are other fast methods, such as Burt’s multi-scale method [33], which can approximate the convolution calculation of a large Gaussian kernel with O(n) time complexity using hierarchical sampling positions. These methods bring more flexible options to the field of image processing while improving efficiency.

In this paper, we introduce a novel multi-scale framework that extends this concept to the 3D level. It is innovative in that it not only effectively approximates a specific kernel but can also be adjusted in O(n) time to simulate the effects of multiple large linear translation-invariant (LTI) filters. The strength of this framework lies in its broad applicability and flexibility to respond to a variety of situations. It not only shows excellent performance in solving Poisson equations for convolution, but also shows excellent applicability in inverse distance-weighted kernels of membrane interpolation and offers generalized support for the Gaussian kernel of dispersion data interpolation.

This method combines a multi-scale strategy similar to the Laplacian pyramid with several wavelet transform techniques. In contrast to traditional transformation, this method is a custom transformation approach, which directly approximates the effect of a given LTI operator and is highly targeted and efficient. It is worth emphasizing that this method is different from the multi-scale structures used in the past. Previous multi-scale approaches often transformed the problem into a more solvable space and then sought solutions in the new space.

We employ an innovative approach to convolution operations with three small fixed-size kernels combined with downsampling and upsampling of images to operate on a variety of scales. This approach looks at numerical optimization of the weights of each kernel so that the overall behavior is closest to performing a convolution operation for a particular target filter. By optimizing each target filter once, the resulting multi-scale transformation can be applied to any input signal in O(n) time, greatly improving the processing efficiency. One of the motivations for designing this approach is to avoid the analytical challenges of dealing with small finite filters. In addition, it makes full use of the linear computing budget to achieve the goal of efficient application of multi-scale operations at a lower computing cost.

#### 2.2.2. Convolution Kernel Training

The optimization process involved in designing the small kernels for the Laplacian pyramid in convolution pyramids allows for the creation of translation-invariant kernels that can effectively capture the essential details across different scales of the image. By modulating the contribution of kernels at each level and optimizing their weights, it is intended to ensure that the multi-scale decomposition would lead to a more accurate representation of the image details at various scales.

By optimizing the kernels to approximate the effect of a specific target filter, the convolution pyramids approach can adapt to the characteristics of the input signal and focus on extracting relevant details that align with the properties of the target filter. This adaptability and customization of the multi-scale decomposition process through learnable kernels can lead to improved detail extraction compared to fixed kernels in traditional Laplacian pyramids.

**Figure 6:**
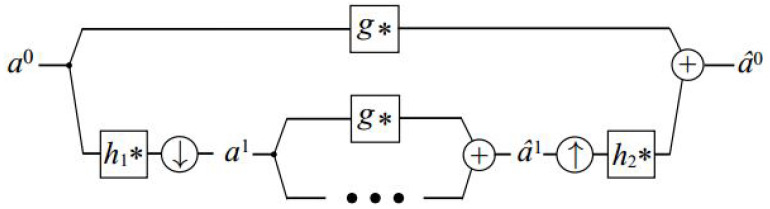
Our sub-band architecture flowchart.

Algorithm 2 and Figure 6 illustrate the multi-scale filtering scheme that we use and which is inspired by the architectures mentioned above in Section 2.2.1. The forward transform includes convolving the signal with an analysis filter $h_{1}$ and the result is subsampled by a factor of two. Then, the process is repeated on the subsampled data. In addition, an unfiltered and unsampled copy of the signal is kept at each level. Formally, at each level, we compute:

(11) $$a_{l}^{0}=a_{l}$$

(12) $$a_{l+1}=\;↓(h_{1}*a_{l}),$$

where the subscript *l* signifies the hierarchy level, $a_{l}^{0}$ represents the unfiltered data retained at each level, and ↓ indicates the subsampling operation. The transformation begins by initializing $a_{0}=a$, where *a* denotes the input signal intended for filtration.
**Algorithm 2** Our multi-scale transform**Input:** Trained filter kernel k=(−0.1820,−0.5007,−0.6373,0.1767,0.5589), divergence of       the video to be reconstructed div;
**Output:** Result video
  1:Filter kernel disassembling: $H=[k_{0},k_{1},k_{2},k_{1},k_{0}]$, $G=[k_{2},k_{3},k_{4}]$$h_{1}=H^{T}H$, $h_{2}=k_{2}\cdot h_{1}$, $g=G^{T}G$  2:Determine the number of levels *L*  3:Forward Transform (analysis):  4:$a_{0}=\mathrm{div}$  5:**for** each level l=0…L−1 **do**  6:    $a_{l}^{0}=a_{l}$  7:    $a_{l+1}=↓\left(h_{1}*a_{l}\right)$  8:**end for**  9:Here get the residual pyramid.10:Backward Transform (synthesis):11:${\hat{a}}_{L}=g*a_{L}$12:**for** each level l=L−1…0 **do**13:    ${\hat{a}}_{L}=h_{2}*\left(↑{\hat{a}}_{l+1}\right)+g*a_{l}^{0}$14:**end for**15:The resulting video is obtained from the pyramid reconstruction.


The backward transformation (synthesis) involves upsampling through the insertion of a zero between each pair of samples. This is followed by convolution using another filter, $h_{2}$. Then, we merge the upsampled signal with the one preserved at that level, achieved by convolving it with a third filter *g*, i.e.,

(13) $${\hat{a}}_{l}=h_{2}*\left(↑{\hat{a}}_{l+1}\right)+g*a_{l}^{0},$$

where the symbol ↑ signifies the zero upsampling operator. It is important to highlight that, unlike in conventional sub-band architectures, our synthesis does not aim to reverse the analysis and reproduce the input signal *a*. Instead, the combined effect of the forward and backward transforms, denoted as ${\hat{a}}^{0}$, is designed to approximate the outcome of a particular linear translation-invariant filtering operation applied to the input *a*.

Among them, the sizes of the convolution kernels $h_{1},h_{2},g$ are very small, usually 3×3×3 or 5×5×5, which ensures the high efficiency of the whole algorithm. When the size of the convolution kernel is small, the speed of convolution calculation is fast. When the size of the convolution kernel is comparable to the length of the signal, the direct convolution calculation requires $O\left(n^{2}\right)$ multiplication, where n is the length of the signal; if the fast DFT or DCT is used, the computational complexity can be reduced to $O\left(n\log n\right)$. The remaining problem is how to obtain the three convolution kernels. In order to approximate convolutions with a given target kernel *f*, we seek a set of kernels $F=h_{1},h_{2},g$ that minimizes the following functional:

(14) $$\begin{array}{c} \underset{h_{1},h_{2},g}{\arg\min}{∥F_{h_{1},h_{2},g}\left(a\right)-f*a∥}_{2} \end{array}$$

This function involves an optimization procedure where the optimization part focuses on minimizing the error metric by adjusting the parameters. Specifically, the goal of the optimization of the procedure is to minimize the difference between the processed video and the original video by adjusting the values of these convolution kernels. In the procedure, the corresponding convolution kernels are computed by adjusting the parameter vectors; then, the video is processed, and finally, the mean value of the image difference is computed. This mean value is used as a target parameter for the optimization which aims to minimize this mean value. The minimization algorithm in GSL (GNU Scientific Library) is used in the program, which provides various optimization algorithms, such as Nelder–Mead, simulated annealing, conjugate gradient, etc., so that the appropriate algorithm can be chosen for parameter optimization according to the situation. The training algorithm is described in Algorithm 3.

This scheme is similar to the discrete fast wavelet transform, with the difference that, as with the Laplacian pyramid, we do not subsample the decomposed signal (the high-frequency band in these transforms and our full band $a_{0}^{l}$). Similar to the high-density wavelet transform [34], this choice is made to minimize subsampling effects and increase translation invariance.

Assuming that ideal filtering is computationally feasible, $h_{1}$ and $h_{2}$ can be chosen to perfectly isolate and reconstruct the lower-frequency bands of the original data, at which point g serves to approximate the desired filtering operation.
**Algorithm 3** Kernel Training Process**Input**: A video for training convolution kernel *k*;      Set a threshold α for the mean deviation between reconstructed video and original      input video;
**Output:** Filter kernel
  1:Set the training kernel parameters to random numbers.  2:Set $a_{0}=\mathrm{div}$  3:Calculate the input video divergence div.  4:**while** $\alpha_{i}<\alpha$ **do**  5:    Disassemble the filter kernel *k*: $H=[k_{0},k_{1},k_{2},k_{1},k_{0}]$, $G=[k_{3},k_{4},k_{5}]$     $h_{1}=H^{T}H=h_{2}$, $g=G^{T}G$;  6:    Rebuild $a_{i}$ to $a_{i+1}$ using Algorithm 2;  7:    Calculate the mean deviation $\alpha_{i}$ between the reconstructed image and the original input image, and update the filter iteratively.  8:**end while**


The idea of iteration is used in kernel training and iterative solutions have many advantages in computing tasks as follows:

They offer flexibility, adapting well to diverse problem settings and allowing for customization to specific requirements; they excel with large-scale problems, efficiently handling systems with numerous variables or data points. Their gradual convergence allows for progress monitoring, enabling computations to halt once a desired level of accuracy is attained, which is particularly beneficial for scenarios tolerating approximate solutions. They necessitate less memory compared to direct methods by updating and storing only a subset of the solution at each iteration, which is ideal for memory-constrained environments and large datasets.

Iterative algorithms effectively parallelize computations, distributing workloads across multiple processors or units and resulting in significant speedups for solving large problems. Their adaptability during the iterative process allows for adjustment based on intermediate results, potentially enhancing efficiency and convergence speed.

However, since we want to keep the number of operations O(n), the filters $h_{1}$, $h_{2}$, and *g* must be finite and small. This means that the design of these filters must take into account this lack of desirability as well as the complex interactions that arise between different frequency bands.

Therefore, instead of deriving filters $h_{1}$, $h_{2}$, and *g* from explicit analytic filter design methods, we numerically optimize these filters so that their joint action best achieves the desired filtering operation. In summary, our approach consists of identifying and allocating a certain number of computations by reducing the amount of subsampling while staying within O(n) computations, and then optimizing the allocated computations to best approximate the convolution using a large filter.

#### 2.2.3. Boundary Interpolation

In this section, we demonstrate a more efficient approach to constructing a suitable membrane by approximating Shepard’s scattered data interpolation method [35] through the utilization of a convolution pyramid.

Consider a region of interest Ω on a discrete regular grid, with boundary values represented by b(x). The objective is to achieve a smooth interpolation of these values across all grid points within Ω. Shepard’s method formulates the interpolant *r* at a point x as a weighted average of the known boundary values.

(15) $$r\left(x\right)=\frac{\sum_{k}w_{k}\left(x\right)b\left(x_{k}\right)}{\sum_{k}w_{k}\left(x\right)},$$

where $x_{k}$ are the boundary points. In our experimental observations, we determined that employing the subsequent weight function yields a satisfactory membrane interpolant:

(16) $$w_{k}\left(x\right)=w\left(x_{k},x\right)=1/d{\left(x_{k},x\right)}^{3},$$

which has a strong spike at the boundary point $x_{k}$ and decays rapidly away from it.

Directly evaluating Equation (15) in a straightforward manner can be computationally expensive. Assuming *K* as the number of boundary values and *n* as the number of points in Ω, the computational cost is O(Kn). Leveraging our multi-scale transform enables us to approximate the computation efficiently in O(n) time. Following Carr et al.’s approach [36], we initially express Shepard’s method in convolution terms. Introducing $\hat{r}$ as an extension of *b* to the entire domain, we have:

(17) $$\hat{r}\left(x_{k}\right)=\left\{\begin{array}{cc} b(x_{k}), & \mathrm{for}\;x_{i}=x_{k}\;\mathrm{on}\;\mathrm{the}\;\mathrm{boundary}\\ 0, & \mathrm{otherwise} \end{array}\right.$$

and rewrite Equation (15) as a ratio of convolutions:

(18) $$r\left(x_{i}\right)=\frac{\sum_{j=0}^{n}w\left(x_{i},x_{j}\right)\hat{r}\left(x_{j}\right)}{\sum_{k=0}^{n}w\left(x_{i},x_{j}\right)\chi_{\hat{r}}\left(x_{j}\right)}=\frac{w*\hat{r}}{w*\chi_{\hat{r}}}$$

The characteristic function $\chi_{\hat{r}}$ corresponds to $\hat{r}$, where $\chi_{\hat{r}}$ equals 1 and where $\hat{r}$ is non-zero and 0 otherwise. This inclusion of the characteristic function χ in the denominator intuitively prevents the accumulation of weights for zeros in $\hat{r}$.

Once again, optimization is employed to determine a set of kernels F for the convolution pyramid’s application to evaluate Equation (18). For training data definition, we assign random values to the boundary grid points of a simple rectangular domain, setting *b* as its boundary. We then calculate an exact membrane r(x) using Equation (18). The optimization process aims to directly match *r*, a ratio of two convolutions, instead of individually matching each convolution. This approach is crucial for accurately reproducing the decay of the filter *w* throughout the entire domain using our transform.

### 2.3. Video Quality Assessment

Video quality evaluation plays an important role in the rapid development of digital video. On the one hand, a large number of video optimization methods have been developed, and on the other hand, video quality evaluation methods are constantly being developed to meet the needs of different scenes.

**Figure 7:**
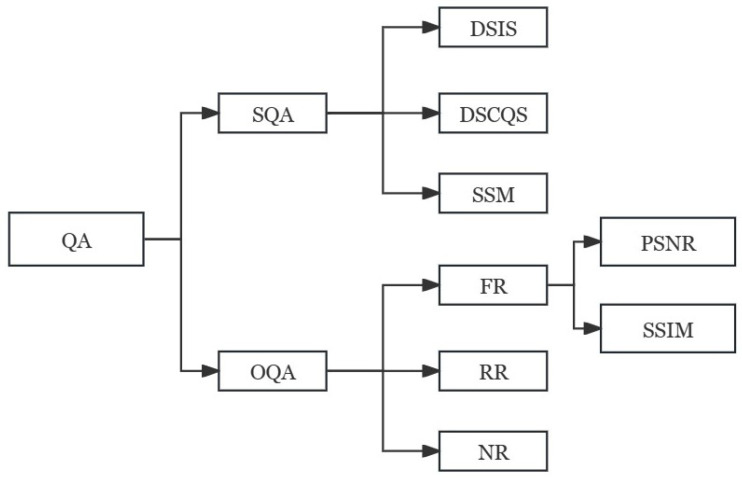
Classification of video quality evaluation.

Generally, digital video quality assessment methods are divided into two categories: video subjective quality assessment (SQA) and video objective quality assessment (OQA). The most accurate evaluation method is the subjective quality evaluation method that lets the viewer directly judge the video quality. However, the method is relatively complex and its results are susceptible to influence by many factors. Therefore, in practical applications, objective and easy-to-implement objective video quality evaluation methods are usually adopted. According to the degree of dependence on the original video, objective quality evaluation methods can be divided into three types: full reference (FR), partial reference (RR), and no reference (NR). The classification of video quality evaluation is shown in Figure 7.

As can be seen from Figure 7, the full reference method is applicable to the encoder end of the known original video, and can be used to quantitatively measure the output video quality of the encoder. It is mainly used for the design of the encoder and the evaluation and optimization of the performance of different encoders. The full reference objective video quality evaluation method refers to comparison between the original reference video and the distorted video in each corresponding frame of each corresponding pixel. Strictly speaking, this method is not the true video quality, but the target video relative to the original video similarity or fidelity degree. The simplest methods, such as the mean square error MSE and the peak signal-to-noise ratio PSNR, are widely used.

The most common full reference video quality assessment methods are as follows:

(1) Mean square error MSE

(19) $$\mathrm{MSE}=\frac{\sum_{0\leq i\leq M}\sum_{0\leq i\leq N}{\left(f_{ij}-f_{ij}^{\prime }\right)}^{2}}{M\times N},$$

where, $f_{ij}$ and $f_{ij}^{\prime }$, respectively, represent the corresponding frame of the original video and the corresponding frame of the target video, and M and N, respectively, represent the height and width of the video frame.

(2) Peak signal-to-noise ratio PSNR

(20) $$\mathrm{PSNR}=10\cdot \log_{10}\frac{{\mathrm{MAX}}^{2}}{\mathrm{MSE}}$$

PSNR is essentially the same as MSE and is a logarithmic representation of MSE.

(3) Structural Similarity Index (SSIM)

This evaluation method based on structural distortion, which means it mainly obtains the final similarity result by comparing the brightness, contrast, and structural changes of two images.

(21) $$\mathrm{SSIM}\left(x,y\right)=l{\left(x,y\right)}^{\alpha }\cdot c{\left(x,y\right)}^{\beta }\cdot s{\left(x,y\right)}^{\gamma }$$

l(x,y)c(x,y)s(x,y) correspond to brightness, contrast, and structural difference functions, respectively.

l(x,y) is measured in average grayscale by averaging the value of all pixels.

(22) $$\begin{array}{c} l\left(x,y\right)=\frac{2\mu_{x}\mu_{y}+C_{1}}{\mu_{x}^{2}+\mu_{y}^{2}+C_{1}}\\ \mu_{x}=\frac{1}{N}\sum_{i=1}^{N}x_{i} \end{array}$$

c(x,y) is measured by the grayscale standard deviation. The unbiased estimate of the standard deviation is:

(23) $$\sigma_{x}={\left(\frac{1}{N-1}\sum_{i=1}^{N}{\left(x_{i}-\mu_{x}\right)}^{2}\right)}^{\frac{1}{2}}$$

Correspondingly, the contrast ratio is expressed as:

(24) $$c\left(x,y\right)=\frac{2\sigma_{x}\sigma_{y}+C_{2}}{\sigma_{x}^{2}+\sigma_{y}^{2}+C_{2}}$$

The structural difference function s(x,y) is the texture and edge information in the image, representing the detail information of the image. Their covariance is compared to measure structural similarity:

(25) $$c\left(x,y\right)=\frac{\sigma_{xy}+C_{3}}{\sigma_{x}\sigma_{y}+C_{3}},$$

where

(26) $$\sigma_{xy}=\frac{1}{N-1}\sum_{i=1}^{N}\left(x_{i}-\mu_{x}\right)\left(y_{i}-\mu_{y}\right)$$

In Equation (19), α,β,γ represent the weight of each indicator, generally 1. When they are all 1, it has:

(27) $$\mathrm{SSIM}\left(x,y\right)=\frac{\left(2\mu_{x}\mu_{y}+C_{1}\right)\left(2\sigma_{xy}+C_{2}\right)}{\left(\mu_{x}^{2}+\mu_{y}^{2}+C_{1}\right)\left(\sigma_{x}^{2}+\sigma_{y}^{2}+C_{2}\right)}$$

## 3. Results

In this section, the proposed method is applied to multiple instances with different numbers of unknown pixels, each consisting of two videos (source and target) and a mask. The resulting fusion video will be compared with the video obtained by the existing method. We use three pieces of video; both of these are simulated by rasterization rendering. One video depicts a plane flying over the ground, and one tracks a flying conical target moving through space. The third piece of video is a high-resolution video showing a view of some cattle and goats grazing on meadows.

Under the same computer configuration, the proposed method and Poisson image editing were applied, and the Poisson fusion based on MVC accelerated the seamless editing of these two videos. Our method involves carrying out 3D Poisson fusion on the image cube composed of two videos, and the other two methods fuse the video frame-by-frame and reassemble it into a video.

**Figure 8:**
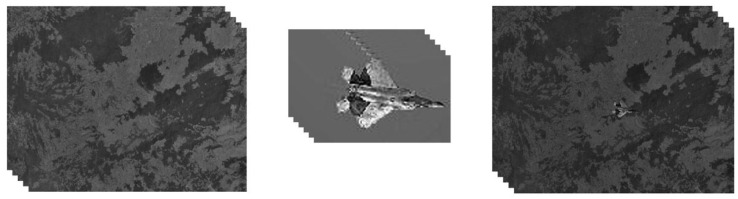
(**Left**) Video of ground surface detected in the air. (**Middle**) Video of a flying airplane. (**Right**) Reconstructed video using our 3D video integration algorithm based on gradient.

**Figure 9:**
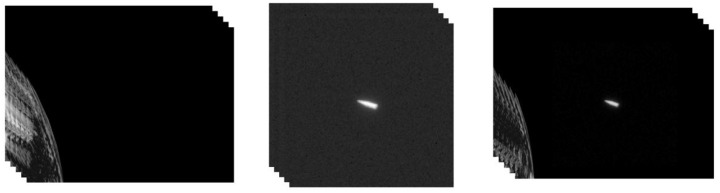
(**Left**) Video of earth in space. (**Middle**) Video of a flying conical target. (**Right**) Reconstructed video using our 3D video integration algorithm based on gradient.

**Figure 10:**
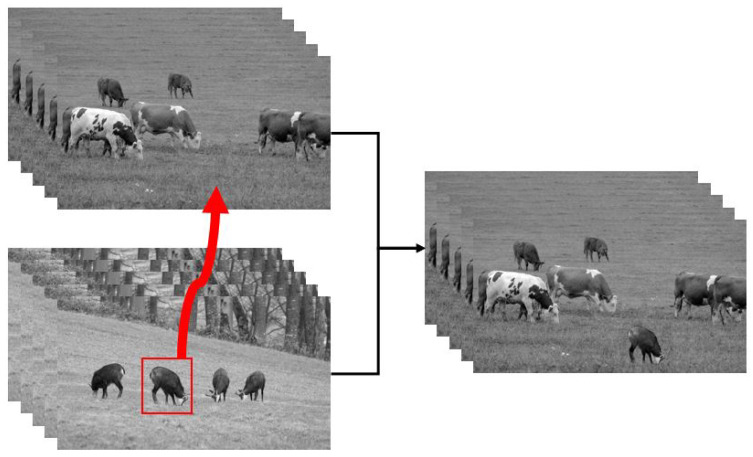
(**Left Up**) Video of cattle. (**Left Down**) Video of goats. (**Right**) Reconstructed video by fusing one of the goats on to the cattle video.

The resolution of the background video of the plane flying video is 640 × 480, and the video resolution of the aircraft target is 100 × 80. The resolution of the background video of the cone flight video is 800 × 600, the resolution of the target video is 512 × 512, and each video has 150 frames. The video editing processes are shown in Figure 8 and Figure 9. The resolutions of the background and target video of the cattle and goat video are both 3840 × 2160, and the fused video has 500 frames. The process is shown in Figure 10.

In the next section, we evaluate the fused video from two aspects. On the one hand, several full-reference objective video quality evaluation methods, namely, the mean square error MSE, the peak signal-to-noise ratio PSNR, and the SSIM evaluation method based on structural distortion, are applied to evaluate the quality of the fused video obtained by the 3D Poisson fusion algorithm and the synthesized video after the frame-by-frame fusion of the existing image fusion algorithms. On the other hand, under the same experimental conditions, the time required to synthesize videos by different methods is statistically analysed and compared. Based on the above two situations, the advantages and disadvantages of the various methods are summarized.

### 3.1. Video Quality Evaluation Using Different Parameters

In this part, we adopt the method of full reference video quality evaluation, and take the video obtained by our proposed 3D Poisson fusion algorithm as a reference to evaluate the other two videos. Based on the equations introduced in Section 2.3, we calculated the quality of video generated by several different video fusion methods five times for each method, and took the average number as the results. The results are shown in Table 1.

It can be seen from Table 1 that we take the video generated by our method as a reference; the MSE value calculated for the other two videos is very close to 0, while the SSIM value is very close to 1. For PSNR, if the calculated value is between 20 and 40, it indicates that the quality between the reference video and the selected video is very close. In summary, several calculated video quality evaluation parameters show that the quality of video generated by 3D Poisson editing is comparable to that generated by the other two methods, but this difference is not easy to see with the naked eye. Therefore, it is necessary to compare the runtime of different algorithms to compare the efficiency of seamless video editing.

### 3.2. Time Cost Measurement

This section calculates the video synthesis time of the methods applied in video seamless editing. The running time of the traditional Poisson image editing method and the MVC-based Poisson fusion acceleration method includes the frame-by-frame fusion of images and the time for synthesizing the fused images into videos. Under the same computer operating environment, we performed the method five times each, and took the average time as their results. The time consumed by these three methods was compared with the time consumed by our proposed 3D Poisson video fusion algorithm, as shown in Table 2.

It is clear that 3D Poisson seamless editing has the fastest running speed of the three algorithms. Increasing the video resolution has a great impact on the algorithm running speed, but the 3D Poisson fusion algorithm still exhibits high efficiency. Considering the two factors of video quality and algorithm running speed, the proposed algorithm not only guarantees the quality of seamless video editing, but also significantly improves the running speed. It is an algorithm worthy of research and application in the field of video editing.

## 4. Discussion

Video synthesis is a very important research area in the field of image and video editing. For existing video synthesis algorithms, the computing speed and the authenticity of the synthesized video still need to be further improved. The existing seamless image fusion algorithm is based on the Poisson image fusion algorithm proposed by Pérez et al. [1] in 2003, and has undergone many improvements in terms of how to reduce the number of iterations in solving the Poisson equation. In this paper, an efficient method is proposed to reduce the time taken in editing videos seamlessly.

We extend the two-dimensional space applicable to the Poisson editing algorithm to three-dimensional space, and expand the usual idea of frame-by-frame image fusion and then stitching into a video. The two videos used to synthesize are treated as three-dimensional image cubes, and then we solve the Poisson equation in three dimensions for these two image cubes using the convolution pyramid method. The algorithm consists of a multi-scale scheme that computes an approximation of linear time through wavelet transform. Given a specific large target filter to approximate, we first use numerical optimization to design a set of small kernels, and then use these small kernels to perform the analysis and synthesis steps of our multi-scale transformation. The main idea of the convolutional pyramid algorithm is that it gradually achieves seamless fusion results by optimizing filters rather than analytically compensating for their lack of idealness. It is suitable for seamless video fusion and is faster than the existing image-editing algorithms.

Although we have demonstrated the effectiveness of this approach in applications, the theoretical analysis of these questions and data remains incomplete, providing possible directions for future work to explore. For example, the video evaluation method we adopted falls within the full reference video quality evaluation approach, which is limited by experimental conditions and technology, while no-reference evaluation is evaluation without any reference to the original video, which is more suitable for online video evaluation, video enhancement, video merging, and other scenarios. In subsequent experiments, this method can be applied to evaluate the generated videos to obtain comparative information. In addition, our work does not clarify what other filters can be approximated using this approach.

Another limitation comes from the black-box optimization used to find the kernel set F. In order to obtain the image cube filter, a three-dimensional kernel must be used. As the number of unknown parameters in an optimization increases, it becomes more difficult and time-consuming to expect the optimization to actually reach a global (or even a satisfactory local) optimal. As future work, we think it is necessary to carry out some theoretical research and mathematical derivations to determine the applicability of this method in terms of filters. If optimization cannot be applied to some filters, at minimum, our research results can contribute to their convergence.

## 5. Conclusions

In this study, we use three sets of videos of different targets and backgrounds as the material to test the seamless video fusion algorithm. We use three different video fusion algorithms, namely, the Poisson fusion algorithm, MVC, and the 3D seamless video fusion algorithm based on a convolutional pyramid, to fuse these two groups of videos, and evaluate the effects of these algorithms by comparing the quality of the fused video and the time of the video editing process.

According to the content of Section 3, the quality gap of the video obtained by the several algorithms is difficult to detect with the naked eye. Taking the fused video obtained by the convolutional pyramid algorithm as a reference, the MSE, SSIM, and PSNR calculated by the Poisson image editing and the mean-value coordinates algorithm show that the video quality obtained by our proposed algorithm is comparable to that obtained by some existing fusion methods, and at the same time, it solves the artifacts and flicker problems caused by frame-by-frame fusion in previous video fusion algorithms. The running time of different algorithms to edit the same video becomes a key factor in evaluation. The results in Section 3 show that for the seamless fusion of two groups of videos, the 3D video seamless fusion algorithm based on a convolutional pyramid has the fastest operation speed.

In summary, the proposed method can be effectively applied to seamless video editing, improving computing speed, and guaranteeing the quality of the fused video.

## Figures and Tables

**Table 1:** Average quality assessment parameters for different videos in comparison to our method.

Method	Cone Target Video			Plane Target Video			Goat Target Video		
	MSE	PSNR	SSIM	MSE	PSNR	SSIM	MSE	PSNR	SSIM
MVC	$4\times 10^{-6}$	53.7657	0.9985	$4\times 10^{-6}$	54.2622	0.9989	$6\times 10^{-6}$	52.01727	0.999530
Poisson	$2.3\times 10^{-5}$	46.3317	0.9971	$8\times 10^{-6}$	51.1269	0.9979	0.00363	34.39736	0.996223

**Table 2:** Average time in seconds of video editing using different methods in seconds.

Method	Cone Target	Plane Target	Goat Target
3D Possion	3.124979	2.023846	171.857758
MVC	14.505209	2.245421	510.760381
Possion	6.597945	2.963986	714.55405

## Data Availability

No new data were created or analyzed in this study.
